# A Run-Length Encoding Approach for Path Analysis of* C. elegans* Search Behavior

**DOI:** 10.1155/2016/3516089

**Published:** 2016-06-30

**Authors:** Li Huang, Hongkyun Kim, Jacob Furst, Daniela Raicu

**Affiliations:** ^1^School of Computing, College of Computing and Digital Media, DePaul University, Chicago, IL 60604, USA; ^2^Department of Cell Biology and Anatomy, Chicago Medical School, Rosalind Franklin University, North Chicago, IL 60064-3095, USA

## Abstract

The nematode* Caenorhabditis elegans* explores the environment using a combination of different movement patterns, which include straight movement, reversal, and turns. We propose to quantify* C. elegans* movement behavior using a computer vision approach based on run-length encoding of step-length data. In this approach, the path of* C. elegans* is encoded as a string of characters, where each character represents a path segment of a specific type of movement. With these encoded string data, we perform *k*-means cluster analysis to distinguish movement behaviors resulting from different genotypes and food availability. We found that shallow and sharp turns are the most critical factors in distinguishing the differences among the movement behaviors. To validate our approach, we examined the movement behavior of* tph-1* mutants that lack an enzyme responsible for serotonin biosynthesis. A *k*-means cluster analysis with the path string-encoded data showed that* tph-1* movement behavior on food is similar to that of wild-type animals off food. We suggest that this run-length encoding approach is applicable to trajectory data in animal or human mobility data.

## 1. Introduction

*C. elegans* is an important genetic model organism relevant to human biology and disease, as its genome is surprisingly similar to that of humans (40% homologous) [1]. Genes encoding tryptophan hydroxylase, which is the key enzyme for serotonin biosynthesis, are conserved in human and* C. elegans* [2]. The biogenic amine serotonin acts in* C. elegans* to modulate behaviors such as egg-laying, pharyngeal pumping, locomotion, and learning in response to changing environmental cues [3]. Variants in human tryptophan hydroxylase are associated with a spectrum of neuropsychiatric disorders, including depression, bipolar disorders, and suicidality [4]. Therefore, by comparing the locomotory behaviors of* C. elegans* wild-type and* tph-1* animals under two different conditions (in the presence and absence of food), we expect to gain insights into understanding how certain genes influence human emotional and congenital disorders.

*C. elegans* locomotory behavior can be recorded and analyzed using computer vision and machine learning approaches. Several worm trackers have been developed to analyze worm behavior in different scenarios such as for recording multiple worms at the same time [5], imaging of specific neurons [6–8], and recording of single animals that are freshly removed from bacterial food, particularly over a long period of time [9]. Video data recorded with the trackers can be further analyzed by quantifying the differences in the worm body and movement characteristics. The differences in these characteristics allow comparing food search behavior across different types of worms and understanding what genetic mutation produces a certain defect in the worm movement. In this study, we (1) quantify the path of a worm as a string of symbols where each symbol represents a segment of a certain type of movement (shallow or sharp turn) and (2) learn the similarities and differences in* C. elegans* locomotory behaviors by comparing their string-encoded path data.

Previous* C. elegans* studies on the neurotransmitter serotonin focus on its role in the regulation of mating, egg-laying, fat storage, reproductive lifespan longevity, and locomotory rate. For example,* C. elegans* males with reduced serotonin levels exhibited defects in tail-curling behavior during mating [10]. A similar observation was made in wild-type males in which serotonergic neurons were ablated. Serotonin signaling in* C. elegans* regulates the function of serotonergic motor neurons that stimulate egg-laying behavior [11]. In particular, serotonin was hypothesized to control a switch between two distinct on/off states of egg-laying behavior [12]. Further, serotonin modulates* C. elegans* locomotory rate in response to bacterial food differently for the well-fed and food-deprived wild-type animals [13]. It is also known that well-fed* tph-1* mutants exhibit changes in behavioral and metabolic processes similar to those caused by starvation: slower rates of egg-laying and pharyngeal pumping, dauer larval arrest, increased fat storage, and an extended reproductive lifespan [2]. Furthermore, a recent study showed that serotonin signaling regulates on food explorative behavior; lack of serotonin signaling increases roaming and decreases dwelling [14]. However, it is still not clear how wild-type and* tph-1* mutant animals compare in off food conditions, particularly with respect to their overall movement paths.

The study of* C. elegans* movement paths is focused on foraging speed, tail motion, crawling, and a specific posture in the movement of the worm. For example, Padmanabhan et al. studied bends during reversing [15], while Gray et al. focused on omega turns [16]. To date, there has been no research focusing on encoding and analyzing the whole path of* C. elegans*. In our paper, we propose to encode the path using run-length encoding, which is a data compression algorithm for sequences of data developed by Duda et al. [17] and extract a set of features that will quantify the characteristics of the path. Run-length encoding has been used in 2D and 3D image texture analysis to quantify texture information and improve image classification accuracy. For examples, Tang [18], Xu et al. [19], and Martinez [20] have also applied run-length encoding in data compression, such as the compression of images, sound, and program code.

Path analysis can be also used to find patterns in human mobility. For example, human flight length can be recorded and calculated from GPS services; Rhee et al. showed that the analysis of the distribution of the flight lengths of a walking path shows that human walk patterns contain statistically similar features observed in Lévy walks [21]. In our paper, by encoding the path with the clustering sequence on three feature variables derived from each video, we are able to generate features for each worm for further analysis. These results can be generalized to human locomotion, as well as technological advances such as self-driving cars.

## 2. Materials and Methods

**Figure 1:**
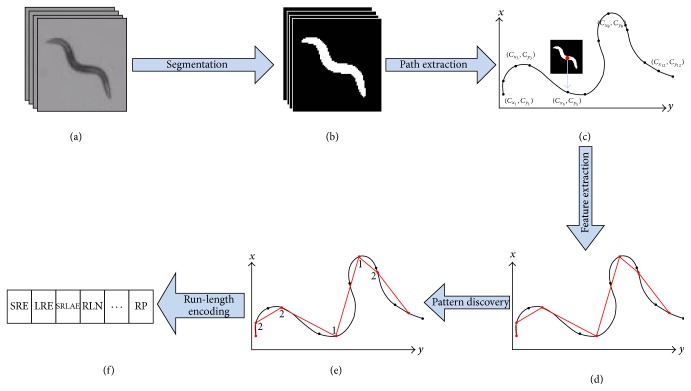
Run-length encoding descriptor extraction: (a) video data for one worm tracking recording; (b) segmented images with worm body pixels shown in white; (c) worm body centroid extraction; (d) step-length, angle, and speed feature extraction; (e) movement pattern discovery (1 and 2 symbols represent the encoding of the path using clustering); (f) extraction of run-length encoding descriptors.

**Figure 2:**
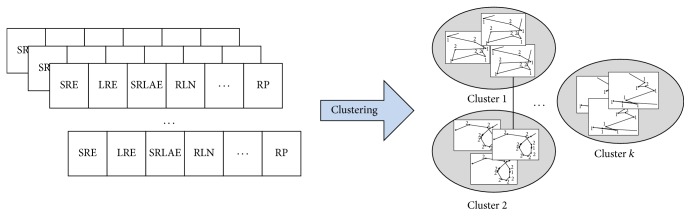
Path similarity: clustering is applied on the worm data in which each video is encoded as a set of run-length encoding descriptors. The result consists of groups of worms that follow similar paths.

Our proposed methodology is based on both computer vision and machine learning components to quantify and compare* C. elegans* locomotory behavior. The raw data consists of image frames from the videos we record with our in-house built worm tracker [9]. The path of each worm is generated from the worm body centroid location in each frame. We propose to (1) encode each path as a string of movement patterns detected using clustering, (2) compress each path string using run-length encoding, (3) extract run-length descriptors that characterize the frequency and order of the detected movement patterns, and (4) use the run-length descriptors to understand the similarities in the locomotory behavior of wild-type N2 and* tph-1* worm data.  Figure 1 illustrates the process of extracting the run-length encoding descriptors and Figure 2 illustrates the path similarity approach based on these descriptors.

### 2.1. *C. elegans* Data and Feature Extraction

Two types of* C. elegans* worm data, N2 and* tph-1*, are collected using our in-house built tracker, which records the movements of single animals at 30 frames per second [9], under three different conditions: on food (N2_f and* tph-1*_f), off food (N2_nf and* tph-1*_nf), and previously starved when placed on a plate without food (N2_nnf; the last condition was experimented for N2 wild-type worms only).  Section 3 presents our findings based on 57 video recordings: 4 N2_f; 33 N2_nf, 2 N2_nnf; 13* tph-1*_f, and 5* tph-1*_nf.

For each video (see example in Figures 1(a) and 1(b)), the following features are extracted based on the worm body centroid position data (*C*_*x*_, *C*_*y*_): step-length *L* (mm), angle ∝ (degree) between two steps, and speed *v* (mm/s) along the step. Rather than working with all the centroid points on the worm path, we divide the path into a series of line segments called “steps” and use the step-length data to quantify the path and infer patterns in search behavior. First, we sample the centroid coordinates of the worm (*C*_*x*_, *C*_*y*_) at a regular time interval Δ*t* = 1 sec forming a dataset of [*t*_*i*_, *C*_*x*_*i*__, *C*_*y*_*i*__] values (Figure 1(c)) where time *t*_*i*_ = Δ*t∗i* and *i* denotes the *i*th point on the sampled path at a time rate of Δ*t*. The sample time interval can be adjusted to balance noise reduction and data fidelity. Second, a “turning event” (TE) is identified at a specific [*t*_*i*_, *C*_*x*_*i*__, *C*_*y*_*i*__] if the current movement heading deviates by more than some threshold angle Θ from the heading at the previous turning event. Studies show that the head angles of wild-type animals moving in a straight direction fluctuate between −30 and 30 degrees. We found experimentally that an angle Θ of 40 degrees is the best tradeoff between the correct sampling of the path and the predictive modeling power of the generated step-length data.

Therefore, a new subsample of centroids (Figure 1(d)) is generated from the sampled path, containing only the centroid locations for the turning events [TE_*i*_, *C*_*x*_*i*__, *C*_*y*_*i*__]. Note that the first centroid location of the worm [*t*_1_, *C*_*x*_1__, *C*_*y*_1__] is considered to be the initial turning event [TE_0_, *C*_*x*_0__, *C*_*y*_0__]. Once the turning events are identified, a step is defined as a line segment between two consecutive turning events [TE_*j*−1_, *C*_*x*_*j*−1__, *C*_*y*_*j*−1__] and [TE_*j*_, *C*_*x*_*j*__, *C*_*y*_*j*__]. The step-length is the displacement between the two turning events forming that step. The angle feature ∝ between two steps is defined as the degree between two movement steps joining three successive turning events, and the speed is calculated by taking the displacement between the two consecutive turning events forming the step and dividing by the interval of time taken to travel that step.

At the end of this feature extraction stage (Figures 1(a)–1(d)), each worm path is represented as a sequence of steps of different lengths that form different angles and are traveled at different speeds. A min-max normalization approach is further applied on the feature data to ensure that all variables have equal importance in determining the patterns along the worm path.

### 2.2. Worm Path Encoding as a String of Movement Patterns

To further quantify the worm path, we investigated the similarity among the steps in terms of their lengths, angles they form, and the speed at which they are traveled. We applied *k*-means clustering approach [17] with the Euclidian distance as the similarity measure and then analyzed the obtained clusters to find insights about any existing pattern characteristics of the worm paths. We varied *k*, the number of clusters, from 2 to 5 and chose the one that generated the best classification results where each cluster was associated with a class of movement patterns. The classification and regression trees (CR&T) approach [22] was applied to validate the choice of *k* using a holdout partitioning to divide the data into training and testing.

Our results showed no difference in the classification accuracy when varying *k* and, therefore, we hypothesize that the path discretized through steps can be simply further encoded as a string of two symbols, where each symbol represents a cluster of a certain movement pattern (Figure 1(e)). A test of significance was then applied to verify the differences between the two clusters with respect to the step data. The angle ∝ between two steps was found to be the most significant in differentiating the clusters (Table 1): cluster “1” was representative for angles smaller than 90 degrees and revealed the “sharp turn” movement pattern while cluster “2” was representative for angles larger than 90 degrees which corresponded to “shallow turn” movement patterns.

Identifying and quantifying these two types of turns are important in a variety of behaviors in the presence and absence of any external stimuli since the worm uses shallow turns to follow sensory information effectively [23–25]. However, only few studies have experimentally and theoretically characterized the shallow turns and quantified their occurrences [26].

### 2.3. Run-Length Encoding Description of* C. elegans* Movement Path

The symbol-based encoded path is further quantified based on a run-length encoding approach [18] that quantifies the frequency of symbols as well as the order in which they appear. For example, for a sequence of symbols “22122221222…,” the run-length encoding will be a sequence of pairs “(2,2)(1,1)(2,4)(1,1)(2,3)….”

Based on the run-length encoding results for each video, a run-length encoding matrix is generated:

(1) $$\begin{array}{c} {P\left(i,j\right)}_{i=1\cdots k,j=1\cdots p}=frequency\left(\;i,j\;\right). \end{array}$$

The rows *i* represent the symbols, columns *j* represent the unique frequencies that symbol *i* shows up in the sequence, and *P*(*i*, *j*) is the count of how many times symbol *i* shows up for *j* consecutive times in the sequence. Matrix *P*(*i*, *j*) can be generated in different ways depending on how the maximum value of *j*, denoted by *p*, is calculated: (1)  *p* is the Maximum Frequency for Individual video data (Matrix IMF); (2) Maximum Frequency across All videos (Matrix AMF); (3) Binned and Maximum Frequency across All videos (Matrix BAMF), and (4) Binned, Averaged, and Maximum Frequency across All videos (Matrix BAAMF).  Table 2 shows an example of these four matrices for one of the worm paths.

The four run-length encoding matrices are* Row 1*: size(IMF) = 2 × 21 (21 is the length of the longest sequence of symbols for one worm path);* Row 2*: size(AMF) = 2 × 37 (37 is the length of the longest sequence of symbols across all worm paths);* Row 3*: size(BAMF) = 2 × 7 (7 is the number of bins in which the lengths were discretized: 1 = 1, 2 = 2, 3 = 3, 4 = 4⋯8, 5 = 9⋯16, 6 = 17⋯32, and 7 = 33⋯37);* Row 4*: size(BAMF) = size(BAAMF) rather than representing *j* as the next available index; here it is represented by the average length within the corresponding bin. The benefit of having AMF versus IMF is that it takes into account the fact that videos have different lengths of time. Furthermore, by binning the lengths, we reduce the sparsity of the run-length encoding matrices as sequences with higher lengths will have less frequency values.

The run-length encoding matrix is further used to extract descriptors using an approach similar to [18] rather than encoding the texture information of an image we propose to encode the frequencies and orders of pattern movements (symbols) of a worm path. The definition of the proposed path descriptors (Table 3) involves four important concepts: short run, long run, low angle level, and high angle level.

*Short run* encoding (SRE) means that the same symbol is repeated shortly and quickly changes into another value;* long run* encoding (LRE) means the same symbol is repeated for a long time before changing into another value;* low angle* encoding (LAE) means the angle between two steps is less than 90 degrees (represented by cluster “1” and defined as a sharp turn); and* high angle* encoding (HAE) means the angle between two steps is more than 90 (represented by cluster “2” and defined as a shallow turn). Using these concepts, we extract eleven path descriptors quantifying the turns and reversals' frequencies and their orders. Table 3 provides the formulas and descriptions where *M* is the number of symbols found using clustering (Section 3.2), *N* is the maximum run length, *n*_*r*_ is the total number of distinct run lengths, and *n*_*p*_ is the number of total step-lengths.

### 2.4. Path Similarity Analysis

Once the path is decomposed into the basic locomotory gaits and transitions between them, the decomposition can be used to understand how animals respond to their environment through movement. In particular, since turning is a fundamental way by which* C. elegans* reorients itself in response to the presence or absence of external stimuli, we propose to use the RLE (run-length encoding) descriptors associated with the symbol-encoded paths to understand how* C. elegans* wild type and* tph-1* behave in the presence and absence of food.

To find the similarities and differences among different search behaviors, we apply *k*-means clustering approach on the* C. elegans* data encoded using the set of eleven RLE descriptors. Given the five different types of experiments (N2_f, N2_nf, N2_nnf,* tph-1*_f, and* tph-1*_nf), we began *k*-means clustering with *k* = 5 and then gradually reduced the value of *k*. The analysis of each cluster will then show which* C. elegans* paths are similar as a function of turn type as well as turn type's frequency and order.

Table 4 shows examples of paths for either low or high values of these descriptors to exemplify how the differences in the descriptor values can help differentiate among different worm paths. Since some of these descriptors are correlated with each other, a data redundancy step is applied on the descriptor data to diminish the effects of this redundancy on the next stage of finding path similarities.

## 3. Results and Discussion

### 3.1. Encoding the Path as a Sequence of Symbols Using Clustering of Step Data

The path was decomposed into steps as described in Section 2.1, resulting in 30,125 steps across all 57 videos for the two types of worms under different food conditions. *k*-means algorithm was applied on the step data (step length, turning angle, and speed along the step) and the number of clusters *k* was varied from 2 to 5 (as described in Section 3.2).  Table 5 shows the characteristics of the clusters for each value of *k*. While the values of the step-length data overlap for length and speed across the clusters for each value of *k*, the angle clearly differentiates between the types of clusters. To validate the clustering results and the selection of *k*, we performed a decision tree classification approach and recorded the accuracy of each of the four classifiers (one classifier for each value of *k*) in predicting the classes associated with the clusters. In all four scenarios, the classification accuracy was 99% meaning that, based on the angle feature, the step data can be clearly divided into 2, 3, 4, or 5 groups of turning angles.

**Figure 3:**
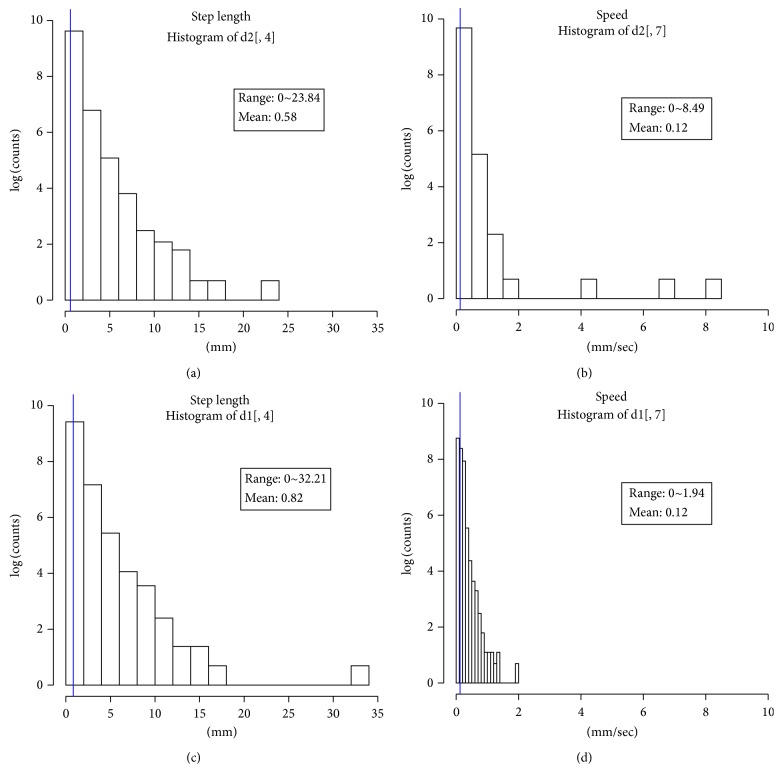
Step data distribution for *k* = 2: (a, b) represent histograms for step-length and speed, for cluster 1; (c, d) represent the same features for cluster 2; the mean value for each cluster is also represented as a thin blue vertical line.

In this study, we decided to choose a coarse level of granularity in quantifying the path movement patterns based on the step-length data by putting the data into two categories (*k* = 2) and show that the two identified turning patterns are important in differentiating behavior in the presence and absence of food. We noticed that when *k* = 2 (Table 5), the two clusters represent two movement patterns, shallow and sharp turns, characterized by angles less than 90 degrees and angles larger than 90 degrees, respectively. In other words, cluster symbol “1” will denote a sharp turn defined as an angle between two consecutive steps being less than 90 degrees and cluster symbol “2” will denote a shallow turn defined as the angle between two consecutive steps being larger than 90 degrees. Within each of the two clusters, although there is a significant overlap between the ranges for speed and length, we noticed that cluster “1” had larger values for its outliers with respect to speed and cluster “2” had larger lengths for its step data (Figure 3). In other words, there are some sharp turns that are executed at higher speeds and some shallow turns that correspond to larger steps.

### 3.2. Run-Length Encoding Descriptors

The symbol-encoded paths of 1s and 2s were further encoded using the run-length encoding approach presented in Section 3.3. Using (1) we generated four run-length encoding matrices (IMF, AMF, BAMF, and BAAMF) and two additional matrices that were the logarithm transformation of the matrices IMF and AMF. For each RLE matrix, we extracted the set of eleven run-length encoding descriptors (using the formulas from Table 3) and analyzed the power of these descriptors in differentiating between N2 and* tph-1* on food and off food. As some of the 11 features are highly correlated, to reduce the redundancy of the data, we performed a correlation analysis for each set of descriptors and kept only the descriptors with correlations smaller than 0.9 for further analysis.  Table 6 shows that only five RLE descriptors can be used further to learn the similarities among the different types of worms and food conditions.

### 3.3. Clustering of* C. elegans* Using RLE Descriptors

The* C. elegans* data was grouped into five clusters using all eleven features and the selected set of features for each one of the six RLE matrices. The analysis of the clustering showed the same groupings for the 57 videos regardless of the calculation of the RLE matrix and the use of all or reduced set of RLE descriptors. Therefore, we present in Table 7 the results for one of the combinations, BAMF matrix and its two selected RLE descriptors, RP and ALN.

The results show that, using the RLE descriptors, we are able to separate 75% of the N2 on food data (cluster 5) from all the other* C. elegans* data. In addition, N2 off food data are relatively well clustered together with* tph-1* off food data in cluster 1 and cluster 3. Most importantly, 100% of the* tph-1* on food data is grouped with 64% of N2 off food data (clusters 3 and 4). Our observation of* tph-1* mutants, which fail to produce serotonin, suggests that* tph-1* mutants continue food search behavior even on food. 100% of the* tph-1* off food data were also grouped with 91% of the N2 off food data across three clusters (clusters 1, 2, and 3). These observations show that* tph-1* mutants either on food or off food behave like wild-type off food animals. Interestingly, through our approach we were also able to distinguish between the two differently collected N2-nnf data, one in which the worm was starved for a longer period of time.

## 4. Conclusions

In this study we aimed to quantify the movement behaviors of wild-type N2 and* tph-1* mutant animals, so that we could identify if* tph-1* mutants show defects in foraging and food search behaviors. By encoding worms' path, extracting features for each worm, and performing clustering analysis, we conclude that the locomotory behavior of* tph-1* on food resembles the wild-type N2 animal off food. In addition, we found that five of the eleven RLE descriptors can be used to efficiently represent the path characteristics for* C. elegans* data. Our unbiased, unsupervised analysis provides evidence that shallow and sharp turns are the most critical factor that distinguishes* C. elegans* movement behaviors in the presence and absence of food. This finding is surprising in that we expected that based on previous studies speed is one of the important factors that determine on food and off food behaviors [13, 16].

As future work, we will investigate the relationships among the* C. elegans* data when encoding the path with more than just two movement patterns. We will look into the cluster patterns formed for larger values of *k* and identify subcluster patterns within the two identified patterns. For the latter, we expect that, for example, if duration of a traveling a step will be important, we can further refine the turns into either short or long turns. We will be also looking into other similarity metrics for the clustering approaches. While we normalized the features individually to the range 0 to 1 using the min-max normalization and then used the Euclidean distance, we have not accounted for the fact that one of the features is an angular component. We will address this limitation by designing a combined similarity metric that will include a cosine similarity metric for the angular component similarity comparisons.

While the RLE descriptors provide important information about the distribution of different path characteristics, the time-component is not integrated in the analysis. To address this limitation, our future work will include the investigation of dynamic time warping (DTW) to compare* C. elegans* paths encoded through sequences of pattern movements. Furthermore, we will be looking into the generalizability of the proposed approach to other domains that make use of trajectory data such as for animal or human mobility studies based on GPS data.

## Figures and Tables

**Figure 4:**
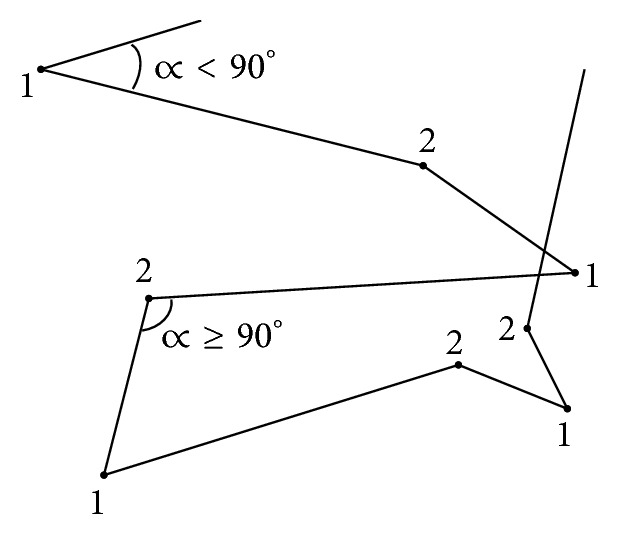


**Table 1:** String symbols definitions resulting from *k*-means clustering (*k* = 2).

Cluster symbol	Cluster name	Significant feature	Movement pattern path encoding
1	Sharp turn	*∝* < 90°	See Figure 4
2	Shallow turn	*∝* ≥ 90°	

**Table 2:** Four run-length encoding matrices.

Matrix	Example (using N2_f1)
IMF	$\begin{array}{cccccccccccccccccccccc} & \text{1} & \text{2} & \text{3} & \text{4} & \text{5} & \text{6} & \text{7} & \text{8} & \text{9} & \text{10} & \text{11} & \text{12} & \text{13} & \text{14} & \text{15} & \text{16} & \text{17} & \text{18} & \text{19} & \text{20} & \text{21}\\ \text{1} & 119 & 71 & 19 & 13 & 5 & 3 & 1 & 0 & 0 & 0 & 0 & 0 & 0 & 0 & 0 & 0 & 0 & 0 & 0 & 0 & 0\\ \text{2} & 76 & 66 & 29 & 20 & 9 & 14 & 6 & 4 & 1 & 1 & 0 & 1 & 2 & 0 & 1 & 0 & 0 & 0 & 1 & 0 & 1 \end{array}$

AMF	$\begin{array}{ccccccccccccccccccccccccc} & \text{1} & \text{2} & \text{3} & \text{4} & \text{5} & \text{6} & \text{7} & \text{8} & \text{9} & \text{10} & \text{11} & \text{12} & \text{13} & \text{14} & \text{15} & \text{16} & \text{17} & \text{18} & \text{19} & \text{20} & \text{21} & \text{22} & \cdots & \text{37}\\ \text{1} & 119 & 71 & 19 & 13 & 5 & 3 & 1 & 0 & 0 & 0 & 0 & 0 & 0 & 0 & 0 & 0 & 0 & 0 & 0 & 0 & 0 & 0 & 0 & 0\\ \text{2} & 76 & 66 & 29 & 20 & 9 & 14 & 6 & 4 & 1 & 1 & 0 & 1 & 2 & 0 & 1 & 0 & 0 & 0 & 1 & 0 & 1 & 0 & 0 & 0 \end{array}$

BAMF	$\begin{array}{cccccccc} & \text{1} & \text{2} & \text{3} & \text{4} & \text{5} & \text{6} & \text{7}\\ \text{1} & 119 & 71 & 19 & 22 & 0 & 0 & 0\\ \text{2} & 76 & 66 & 29 & 53 & 6 & 2 & 0 \end{array}$

BAAMF	$\begin{array}{cccccccc} & \text{1} & \text{2} & \text{3} & \text{6} & \text{13} & \text{22} & \text{36}\\ \text{1} & 119 & 71 & 19 & 22 & 0 & 0 & 0\\ \text{2} & 76 & 66 & 29 & 53 & 6 & 2 & 0 \end{array}$

**Table 3:** Run-length encoding descriptors.

Formula	Description
$SRE=\frac{1}{n_{r}}\overset{M}{\underset{i=1\;\;}{\sum }}\overset{N}{\underset{j=1}{\sum }}\frac{P\left(i,j\right)}{j^{2}}$	Short run encoding (SRE) measures the distribution of short runs

$LRE=\frac{1}{n_{r}}\overset{M}{\underset{i=1\;\;}{\sum }}\overset{N}{\underset{j=1}{\sum }}P\left(i,j\right)*j^{2}$	Long run encoding (LRE) measures the distribution of long runs

$LARE=\frac{1}{n_{r}}\overset{M}{\underset{i=1\;\;}{\sum }}\overset{N}{\underset{j=1}{\sum }}\frac{P\left(i,j\right)}{i^{2}}$	Low angle run encoding (LARE) measures the distribution of the small angles

$HARE=\frac{1}{n_{r}}\overset{M}{\underset{i=1\;\;}{\sum }}\overset{N}{\underset{j=1}{\sum }}P\left(i,j\right)*i^{2}$	High angle run encoding (HARE) measures the distribution of the large angles

$SRLAE=\frac{1}{n_{r}}\overset{M}{\underset{i=1\;\;}{\sum }}\overset{N}{\underset{j=1}{\sum }}\frac{P\left(i,j\right)}{i^{2}*j^{2}}$	Short run low angle encoding (SRLAE) measures the joint distribution of short runs and sharp turns

$SRHAE=\frac{1}{n_{r}}\overset{M}{\underset{i=1\;\;}{\sum }}\overset{N}{\underset{j=1}{\sum }}\frac{P\left(i,j\right)*i^{2}}{j^{2}}$	Short run high angle encoding (SRHAE) measures the joint distribution of short runs and shallow turns

$LRLAE=\frac{1}{n_{r}}\overset{M}{\underset{i=1\;\;}{\sum }}\overset{N}{\underset{j=1}{\sum }}\frac{P\left(i,j\right)*j^{2}}{i^{2}}$	Large run low angle encoding (LRLAE) measures the joint distribution of large runs and sharp turns

$LRHAE=\frac{1}{n_{r}}\overset{M}{\underset{i=1\;\;}{\sum }}\overset{N}{\underset{j=1}{\sum }}P\left(i,j\right)*i^{2}*j^{2}$	Large run low angle encoding (LRHAE) measures the joint distribution of large runs and shallow turns

$ALN=\frac{1}{n_{r}}\overset{M}{\underset{i=1\;\;}{\sum }}{\left(\overset{N}{\underset{j=1}{\sum }}P\left(i,j\right)\right)}^{2}$	Angle level nonuniformity (ALN) measures the similarity of angle level distributions

$RLN=\frac{1}{n_{r}}\overset{N}{\underset{j=1\;\;}{\sum }}{\left(\overset{M}{\underset{i=1}{\sum }}P\left(i,j\right)\right)}^{2}$	Run length nonuniformity (RLN) measures the similarity of run length distributions

$RP=\frac{n_{r}}{n_{p}}$	Run Percentage (RP) measures the homogeneity of the distribution of runs

**Table 4:** Path sequences that illustrate different values for the RLE descriptors.

Example of paths	Description
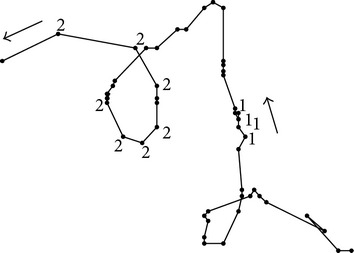 Path subsequence: 1 1 1 1⋯2 2 2 2⋯2 2	Small Short Run Emphasis (SRE) Large Long Run Emphasis (LRE)

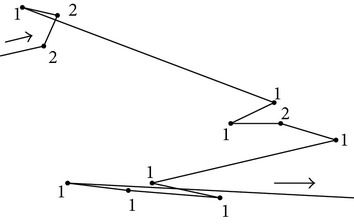 Path subsequence: 2 2 1 1 1 2 1 1 1 1 1	Large Short Run Emphasis (SRE) Small Long Run Emphasis (LRE) Large Low Angle-Level Run Emphasis (LARE) Small High Angle-Level Run Emphasis (HARE)

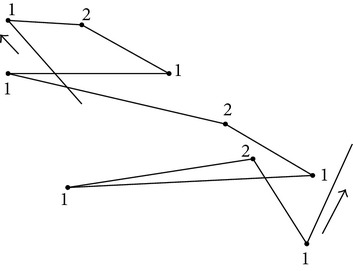 Path subsequence: 1 2 1 1 2 1 1 2 1	Small Short Run High Angle-Level Emphasis (SRHAE) Large Long Run Low Angle-Level Emphasis (LRLAE)

**Table 5:** Step data ranges for clustering results when the number of clusters *k* is varied from 2 to 5.

Number of clusters	Cluster ID	Angle (degree)	Step length (mm)	Speed (mm/s)
*k* = 2	Cluster 1	0~84.32	0~23.84	0~8.49
	Cluster 2	82.64~180	0~32.21	0~1.94

*k* = 3	Cluster 1	0~53.08	0~23.84	0~8.49
	Cluster 2	52.16~116.87	0~32.21	0~6.56
	Cluster 3	116.2~180	0~16.84	0~1.34

*k* = 4	Cluster 1	0~35.91	0~23.84	0~1.48
	Cluster 2	126~180	0~16.84	0~1.32
	Cluster 3	35.34~77.21	0~17.87	0~8.49
	Cluster 4	77.21~126.43	0~32.21	0~1.94

*k* = 5	Cluster 1	0~31.29	0~23.84	0~1.48
	Cluster 2	146.1~180	0~16.84	0~1.32
	Cluster 3	106.3~146.1	0~15.65	0~1.34
	Cluster 4	68.02~107.15	0~32.21	0~1.94
	Cluster 5	30.46~68.04	0~17.87	0~8.49

**Table 6:** Selected feature for each type run-length encoding matrix.

Type of RLE matrix	Selected descriptors
IMF	RP, LRHAE, ALN, SRHAE
AMF	RP, LRHAE, ALN
BAMF	RP, ALN
BAAMF	RP, LRHAE, SRHAE
IMFL	RP, SRE, ALN
AMFL	RP, ALN

**Table 7:** Cluster makeup based on RLE descriptors; each cell number represents the ratio between the number of worms of a specific type that fall under that cluster and the total number of worms in that cluster; the numbers in parentheses represent the ratio between the number of worms of a certain type which fall under that cluster and the total number of worms of that type.

		Cluster 1	Cluster 2	Cluster 3	Cluster 4	Cluster 5	Total # of videos
*C. elegans* data	N2_f	0	0	0	0.12 (25%)	**1 (75**%**)**	4
	N2_nf	**0.84 (33**%**)**	**0.5 (3**%**)**	**0.58 (55**%**)**	**0.38 (9**%**)**	0	33
	N2_nnf	0.08 (50%)	0	0.03 (50%)	0	0	2
	*tph1_*f	0	0	**0.29 (69**%**)**	**0.5 (31**%**)**	0	13
	*tph1*_nf	**0.08 (20**%**)**	**0.5 (20**%**)**	**0.10 (60**%**)**	0	0	5
